# The evolutionary accessibility of cultural fitness landscapes

**DOI:** 10.1371/journal.pcbi.1014830

**Published:** 2026-10-22

**Authors:** Laurel Fogarty, Rebecca B. Hoyle

**Affiliations:** 1 Department of Human Behavior, Ecology, and Culture, Max Planck Institute for Evolutionary Anthropology, Leipzig, Germany; 2 School of Mathematical Sciences, University of Southampton, Southampton, United Kingdom; Interdisciplinary Transformation University IT:U, AUSTRIA

## Abstract

Many human cultural and technological traits have a complex and peculiar structure. They are often characterized by multiple closely interacting parts that can form modules, and change as a result of innovation, collaboration, and social learning. A full understanding of the ways in which such cultural traits evolve requires a model that accounts for the close epistatic interaction of the constituent parts, and for the uniquely cultural processes that act on them. Here, we generalize the concept of ‘adaptive landscapes’, where populations search a landscape for optimal combinations of components. This generalization accounts for epistatic interactions, and includes processes such as modularization, communication, and collaboration, that are not features of genetic systems but may be quite common features of cultural systems. We show that modularization in combination with collaborative communication of search results is more successful, but sometimes more costly, than searching a full adaptive landscape. However, in many of the circumstances under which technology evolves, a full search of many thousands of possible modules may not be feasible since time, financial, energetic, and environmental constraints are likely to be strong. Under these conditions, our models suggest that there is a critical value for the number of modules to search below which the global optimum is more likely to be reached by searching the full landscape. The value of this threshold depends on the complexity of the technology. Overall, we suggest that analysis of cultural fitness landscapes is likely to be a fruitful avenue for research into technological change especially when combined with elements of human learning and interaction known to be important features of cultural evolution.

## Author summary

How human technology changes and evolves, and what forces drive its evolution are under-explored. We generalize the idea of a fitness landscape for cultural rather than genetic traits and ask whether uniquely cultural processes such as collaboration and communication can change the accessibility of cultural fitness landscapes. Technological traits share many broad structural features with the genes or proteins more typically modeled this way. For example, both are composed of many interacting interdependent parts, and can be characterized by considerable fitness epistasis. However, cultural traits are also subject to some quite different forces. When populations search a cultural landscape for optimal technologies, they can do so by deliberately restricting their search to particular parts or modules of the technology, and by collaborating with others seeking to improve other parts of the same technology. We show that this kind of modularization and collaboration makes fitness landscapes easier to search and makes populations more successful at doing so.

## Introduction

Generalisations of genetic evolutionary theory permit investigation into a number of systems that evolve but are not genetic. One example of such a system is human culture [1–3]. The field of cultural evolution views culture as an evolutionary system of inheritance, cultural innovation, cultural selection, and drift and aims to understand the guiding principles underlying human cultural and technological change. This means that many of the modelling frameworks and approaches used to understand genetic evolution can be adapted for use in models of cultural change [4]. Here we propose to generalise the mathematics of evolutionary ‘adaptive landscapes’ [5] to include processes, such as modularisation, communication, and collaboration, that are not features of genetic systems but may be quite common features of cultural ones.

### Fitness landscapes

Adaptive evolution has been described as a complex combinatorial optimization process [6] and the analogy of adaptation as movement on a ‘fitness landscape’ has been used extensively in evolutionary biology since its introduction by Wright in the 1930s [7]. In this ‘mutational pathway’ metaphor, populations move step-wise from one genotype to a single-mutant neighbouring genotype provided that genotype is of higher fitness. Thus, a population can move across a fitness landscape towards fitness peaks (characterised by high-fitness genotypes) and out of fitness troughs (characterised by low-fitness genotypes) to, perhaps, reach a global fitness optimum.

This forms the basis of Wright’s ‘shifting balance theory’ (SBT) of adaptive evolution [7]. The SBT entails three phases. In phase (I) local populations lose fitness as genetic drift causes them to fall into, and cross, “adaptive valleys.” Phase (II) entails that these local populations begin to move towards new “adaptive peaks,” or in other words adaptive phenotypes spread and fix within these local populations. Finally, in phase (III) sub-populations which are positioned on different fitness peaks compete, and the highest fitness phenotype spreads through migration and overtakes the meta-population. According to the SBT, therefore, the spread of high fitness phenotypes in genetic systems proceeds through direct competition.

The fitness landscape best describes evolution in systems where epistasis is common and small mutational changes are rare and likely to occur one at a time. Lower fitness mutations are unlikely to fix and so such mutations limit movement on the landscape. The fitness effect of particular mutations determines the shape of a landscape and the ease with which it can be traversed. For example, landscapes are ‘rugged’ and hard to cross when a single point mutation in the gene under consideration has large or unpredictable fitness consequences [8–11]. Conversely, ‘correlated’ landscapes imply that single mutant neighbours have correlated fitness values. This yields smoother landscapes that can be easier to cross. The probability that at least one pathway to the global optimum exists is often of interest and can be calculated or, in some more complex cases, inferred by simulation (see, e.g., [12–14]).

The results of such calculations and simulations rest on important assumptions about the physical constraints of the genetic system, and so may not generalise beyond the scope of genetic or molecular evolution. Cultural or technological traits may evolve in ways that are broadly similar, or analogous to, genetic evolution [1,3]. However, in the context of fitness or adaptive landscapes, there may be fewer constraints on the processes that allow a population to reach a global optimum. Here, we aim to address the possibility that, in culturally evolving systems, the search through landscapes and the spread of high fitness types might proceed in a number of ways that are unavailable to genetic systems. We expand on these possibilities below.

### The evolution of technology

Cultural evolution differs from genetic evolution in several important respects. Here, we focus on the following: First, cultural evolution can be driven by more deliberate or directed processes than its genetic counterpart. For example, intelligent modularisation and deliberate innovation may play a substantial role in cultural evolution (see [15] for a discussion of this point). And second, ideas, information, or technological components may be communicated and recombined, producing novel traits and combining insights from searches in different parts of fitness landscapes.

Modularity has been identified as an important and perhaps fundamental structural feature of many human technologies [16–18], meaning that many technologies consist of simpler sub-units that can be tested, analysed, maintained, and changed independently. This makes technologies more robust and repairable [16,18] but also means that a search for technological improvement in many cases may not be best modeled as a search on a single ‘fitness landscape’ [19] but rather a number of simultaneous searches on parts of landscapes representing improvement in distinct modules. It has also been suggested that recombination may be characteristic of human culture more generally and, further, that the exponential increase in the ‘amount’ of culture in various domains (for example the abundance of new contributions to a scientific discipline) relies on a feedback between culture and innovation [20] – more culture, more possible novel combinations.

Thus, when crossing *cultural* fitness landscapes, sub-populations might be able to modularize the landscape, in other words, search a small sub-section of a complete landscape, communicate and combine their innovations with those of other sub-populations searching other parts of the landscape and thus reach a global optimum that might otherwise be difficult or impossible to reach. This process bears some similarity to the SBT (described very briefly above) in which sub-populations climb sub-optimal fitness peaks and can then compete with one another. Where Wright’s biologically grounded idea relies on naturally arising population structure alongside non-zero rates of migration and gene flow, our proposed cultural mechanism is slightly different. Instead, the required subdivision of the populations and the landscapes relies on readily demonstrable human problem-solving abilities, and the spread and recombination of sub-optimal components relies on the widespread human capacity for collaboration and communication [21,22].

To give one example of what such a fitness landscape might look like in a cultural context, we consider a widespread but varied indigenous technology: cassava detoxification. Cassava (*Manihot esculenta*) is an important drought resistant tropical root crop which represents a staple food and a source of emergency nutrition for over 500 million people worldwide. However, the presence of severely toxic cyanogenic glycosides (specifically linamarin and lotaustralin) in cassava roots and leaves limits its use and poses a serious risk to human health. If not properly processed cassava causes acute intoxication, neurodegenerative disorders, or death [23,24]. Many complex detoxification procedures have been developed by indigenous communities throughout South and Central America, Africa, and Asia. The full range of such techniques employed by communities around the world, how they have evolved, how effective they are or can be [25,26], and how they spread [27] are of global importance encompassing issues of public health and food security. Most traditional processing techniques consist of a number of steps and often include a subset of the following components: peeling, grating, leaching, pounding, fermenting, boiling, and sun-drying [28,29]. The effectiveness of many of these techniques has been studied chemically and it is possible to piece together a ‘fitness landscape’ consisting of some of these component processes (Fig 1) where ‘fitness’ is assigned according to the effectiveness of detoxification associated with the technique (see Table 1 and below). In Fig 1, red arrows denote accessible paths with monotonically increasing ‘fitness’ values and grey arrows indicate paths in which equal or lower fitness values are encountered. We note that many fitness landscapes could be constructed from the same data depending on the components present, the order in which processes are performed, details of the processes (for example, length of soaking), as well as cultural context. However, we present one simple landscape consisting of four simple processes for the sake of demonstration. Real cultural fitness landscapes are likely to be beset by additional complicating factors such as the possibility of large mutational steps or context-dependent fitness values.

**Fig 1:**
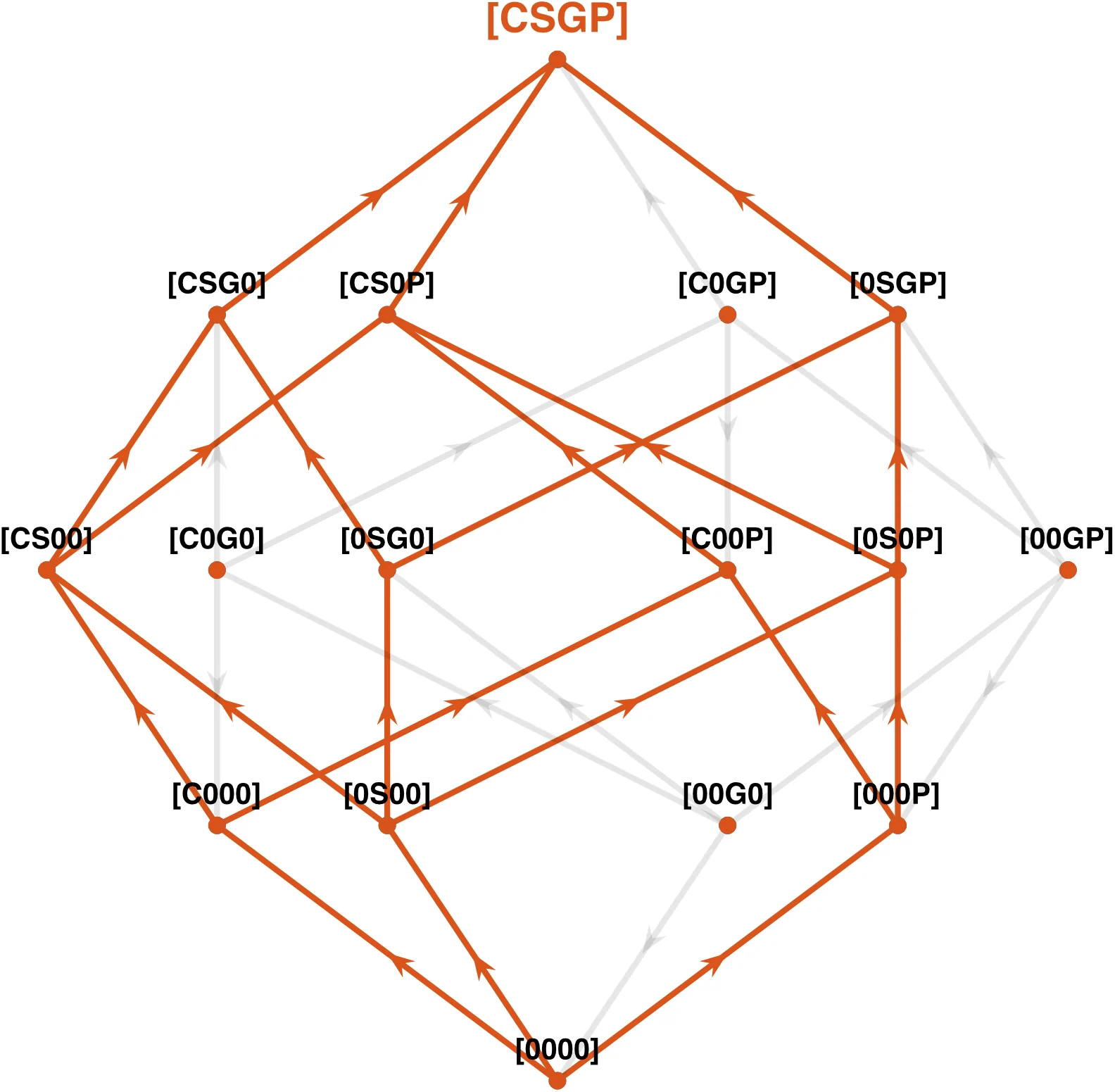
A graphical representation of one (of many possible) cultural fitness landscapes constructed from information about the effectiveness of different simple cassava detoxification processes (see [28]) and Table 1. The components are cooking **(C)**, soaking **(S)**, grating **(G)**, and peeling **(P)**. The ‘fitness value’ of a node is given by the effectiveness of the technique represented at that node. At **[0000]**, none of the processing components are present, the cassava has not been processed and is toxic. At the node **[CSGP]** individuals process cassava using a combination of all simple processes. There is one optimum but not all paths are accessible. Red edges show accessible paths to that optimum.

**Table 1:** Construction of the cassava preparation fitness landscape (Fig 1).

Component	Notes	Value
Peeling	“When tissue types were compared, the concentration of cyanide was 3.3 times greater (when pooled across cultivars) in the tuber peel than the tuber parenchyma.” [41]	0.5 (lower bound)
	“On average, the concentration of cyanide was 5.6 times higher in the peel than the flesh across all cultivars and both sites.” [41]	
	“The concentration of cyanide in the tuber peel was much higher (an order of magnitude for some cultivars) than that of the tuber parenchyma and leaves.” [41]	
	“By peeling the root, 50% of the cyanogenic glycosides in the root can be eliminated.” [28]	
	“More than 50% of the cyanide present in the root is concentrated in the peels” [42]	
Cooking (boiling, 25 minutes)	“The [cassava] chips were thoroughly cooked after 25min, the dry matter content had decreased from about 25 to 19% and 55% of the bound cyanide content had been removed.” [43]	0.45 (lower bound)
	Fig 3 in [43] shows removal of just under 50% of total original cyanide content after 25 minutes	
	“ An early report on the effect of boiling on the cyanogenic glycoside content of cassava revealed that boiling for 25 minutes decreased bound cyanogenic glycoside by 45 to 50%” [28]	
Soaking (24 hours)	[26] reports a controlled experiment comparing soaking of peeled and unpeeled cassava roots for between 24 and 48 hours.	0.31
	“In both the parenchyma and peelings, major drops occurred after 24 h: 14.6% and 31.0% reductions were observed in the peelings and the pulp respectively.” [26]	
Grating	“Although grating alone is insufficient for detoxification, it can be used with other procedures to improve HCN evaporation or reduction via fermentation” [28]	0 when alone (lower bound)
	“Grating only is not sufficient for detoxification, but can be combined with other methods to enhance HCN evaporation or reduction by fermentation [29]	
	“The most important processing stages, with regard to elimination of cyanide, were the initial grating of the cassava and the final roasting of the product” [25]	
	“During the grating or mincing of cassava, the considerable tissue cellular disruption results in greater contact between the endogenous linamarase and its substrate, linamarin [25]”	
	Fig 1 in [25] shows a decrease in total cyanide from around 170–152 parts per million (dry weight) after 1 hour during which the cassava was grated falling to around 75 ppm after 20 hours with pressing, implying that grating with pressing has an effect (around 55% reduction in 20 hours).	
	[44] report that grating alone leaves 1375.40 mg/kg of total cyanide in bitter cassava, which is reduced by pressing.	

The benefits and costs of modularizing adaptive landscapes and combining the results of partial searches of fitness landscapes in this way are far from clear and may be counter-intuitive. For example, if we consider the landscape to be a directed graph with links to accessible mutational neighbors being the only permissible edges (see modeling sections below), connected components of a sub-graph may lead to local optima which cannot be recombined to produce the global optimum. Thus, these new processes may alter, for better or worse, the ‘accessibility’ [13,30] of cultural fitness landscapes compared to almost identical genetic landscapes. In other words, modularization may alter a population’s ability to reach a global optimum, or the most efficient technology. Often, the production of human technology (or any cultural objects) is time- or resource-constrained [31,32]. Here, too, modularization may change how populations traverse a landscape. Simultaneous searches on different parts of a landscape might reduce the time and perhaps even resources that must be expended to reach an optimal technology, and experimental evidence suggests that this ability is key to human cumulative culture [33]. Below, we take one approach to understanding what the ability to modularise and search sub-landscapes might do to enhance or hinder human technological searches, but note of course that many others are possible.

We focus on processes that characterize cultural or technological landscapes and propose a series of cultural fitness landscape models designed to examine the potential costs and benefits of modularizing complete fitness landscapes, searching these modules, and recombining (or, equivalently, communicating) the results. We draw on existing empirical examinations, mathematical and simulation models of genetic fitness landscapes (e.g., [6,13,30,34–36]) and on work that deals with the slightly different case of genetic recombination and fitness landscapes (e.g., [14,37]). In particular, we focus on the probability of crossing a fitness landscape, the time taken to do so, and the communication and search costs involved in both modularized and full fitness landscapes. We discuss the potential implications of these models for our understanding of the role of recombination and collaboration in technological innovation and cultural evolution more generally.

## Materials and methods

### Cassava detoxification example

Following the example described above, we select four possible components of a ‘cassava detoxification’ fitness landscape: peeling (P), cooking (C), soaking (S) and grating (G). We construct the fitness landscape for this cassava preparation example using the information in Table 1. The resulting landscape is shown in Fig 1. In order to assign fitness values to nodes in Fig 1 we make the following assumptions: First, a method with value *v* removes (v*100 of cyanide present in the cassava at the start of that processing step. And Second, methods proceed in the following intuitive order when present: peeling, grating, soaking, cooking.

Although we could find no direct data on the grating step, we followed the following logic: soaking/leaching of whole or large pieces of cassava root in fresh water is effective in reducing both bound and free HCN [26]. However, a large amount of bound HCN appears to remain after soaking for 24 hours [38]. report that increasing disruption of the cell walls allows linamarin to make contact with endogenous linamarase producing volatile HCN, which can be more easily removed by soaking in a slurry. Ultimately, their study suggests that cell-wall disruption led to a reduction of residual cyanide in cassava products. Grating causes mechanical disruption of the cell walls and therefore we assume that grating can increase the value of soaking. Here, for the sake of illustration, we assume that grating increases the effectiveness of soaking by 0.1. We note that, in general, the effect of grating is nuanced (e.g., it is thought to decrease the effectiveness of fermentation [39] but increase the effectiveness of drying [40]) and we stress that the values we assign are for demonstration.

### The fitness landscape models

We examine the dynamics of adaptation of a population where each individual possesses a cultural complex consisting of *N* component parts. This is the cultural equivalent of a genetically haploid, asexually reproducing system of *N* binary genetic loci, each taking a value of either 0 or 1. Here we use the term ‘component’ in the cultural case and use ‘locus’ only in a genetic context. We assume that innovations are relatively rare so that technological change proceeds one innovation or change at a time. In other words, paths from a sub-optimal to an optimal technology proceed from the current type to a single-change neighbour at each step. This is analogous to the assumption of strong selection and weak mutation in similar models of genetic change on a fitness landscape. Fig 1 shows an example of a cultural landscape (the cassava preparation example) where *N* = 4 from an initial state assumed to be **[0000]**, which is the global fitness minimum, to the global fitness maximum, which is **[CSGP]**.

We are interested in calculating the number of such paths that are ‘accessible’, in other words, how many paths exist between the initial and the optimum trait configuration where each step increases the fitness value, bringing the population closer to the optimum. This number depends on the rank order of fitness values of the intermediate forms. It is this ranking that determines the structure of the fitness landscape and the most efficient ways to traverse it. Here we consider the House of Cards (HoC) model [8], where fitness values are uncorrelated and each fitness value can occur just once.

The highest fitness is assigned to a type, which here we denote all ones **[11 ...1]**. In the constrained HoC model, the lowest fitness is assigned to the ‘antipodean’ type (so, all zeros) [45]. In the unconstrained House of Cards model the ‘all zeros’ type is not constrained to have the lowest fitness.

Some immediate results follow from the construction of the landscapes. For example, for the unconstrained HoC model, there are *N*! possible paths from the initial state **[00 ...0]** to the most fit type **[11 ...1]** and each of those paths contains N−1 intermediate types. Fitness is randomly assigned to each of the types other than the global optimum, and each fitness value occurs just once, meaning that just one arrangement out of a possible *N*! along each path is monotonically increasing. This means that in this model there is an expected


$$\begin{array}{c} N!\times \frac{1}{N!}=1 \end{array}$$

accessible path through the landscape, for all *N* [13]. This is an expected value, and the number of such accessible paths that exist in any particular realization of this type of landscape can vary, and may be 0. Therefore, we are also interested in the probability that an accessible path to the global optimum exists through a HoC landscape.

The number of steps in each shortest path (and so the time taken to reach the optimum) can be compared between modular and non-modular cases. Assuming that searches on modular landscapes are simultaneous, the number of steps required to reach the optimum in the non-modular case is *N* and in the modular case is given by the maximum module size. In other words, it is possible that the optimal solutions could be found, and the approach to that optimum could be quicker when the technology is modular. For different landscapes, the relationship between the number of components, *N*, and the probability of finding an accessible path is not, in general, monotonic [13]. This implies that whether modular technology confers benefits in terms of the efficiency of innovation and search for an optimum depends on the landscape on which it is evolving, the number of its constituent parts, and the costs of searching and communication.

### Decomposing a fitness landscape

The total number of possible modules and the total number of different decompositions of a particular landscape can be calculated by treating the technology’s constituent parts as a list of components. We assume that each landscape of *N* parts is decomposed without overlaps apart from the initial state, into disjoint module landscapes each of $n_{i}$ parts such that $\sum_{i}n_{i}=N$.

The number of possible decompositions can be calculated as the total number of ways a set of *N* elements can be partitioned into non-empty, non-overlapping subsets. This is called the ‘Bell number’, often denoted $B_{N}$ [46]. Bell numbers can be calculated as the sum of the relevant Stirling numbers *S*(*N*,*k*), which describe the the total number of ways to partition a set with *N* entries into exactly *k* non-overlapping, non-empty subsets (see [47] for more mathematical details). Stirling numbers are defined using the following recurrence relation, with *N* denoting the total number of elements in a set and *k* the number of subsets:


$$\begin{array}{c} S(N,k)=kS(N-1,k)+S(N-1,k-1). \end{array}$$ (1)

So, if the technology we are considering has *N* component parts, it is possible to calculate the total number of possible partitions or, if the desired number of modules is known, we can calculate the total number of decompositions of that kind. For example, suppose that the evolving technology has 3 components so the landscape has the structure **[a b c]**. The technology can be modularized (or decomposed) into three possible non-empty, non-overlapping decompositions: **[[a],[b c]]**, **[[a b],[c]]** or **[[a c],[b]]**, when *k*, the number of modules is given by *k* = 2.

We assume that the fitness of each node in the module landscape is given by the fitness of the corresponding node in the full landscape with all parts not in the module landscape treated as if they are absent. Other choices could be made, and the implications of alternative choices could be investigated. An example of a HoC landscape with modularisation is given in Fig 2.

**Fig 2:**
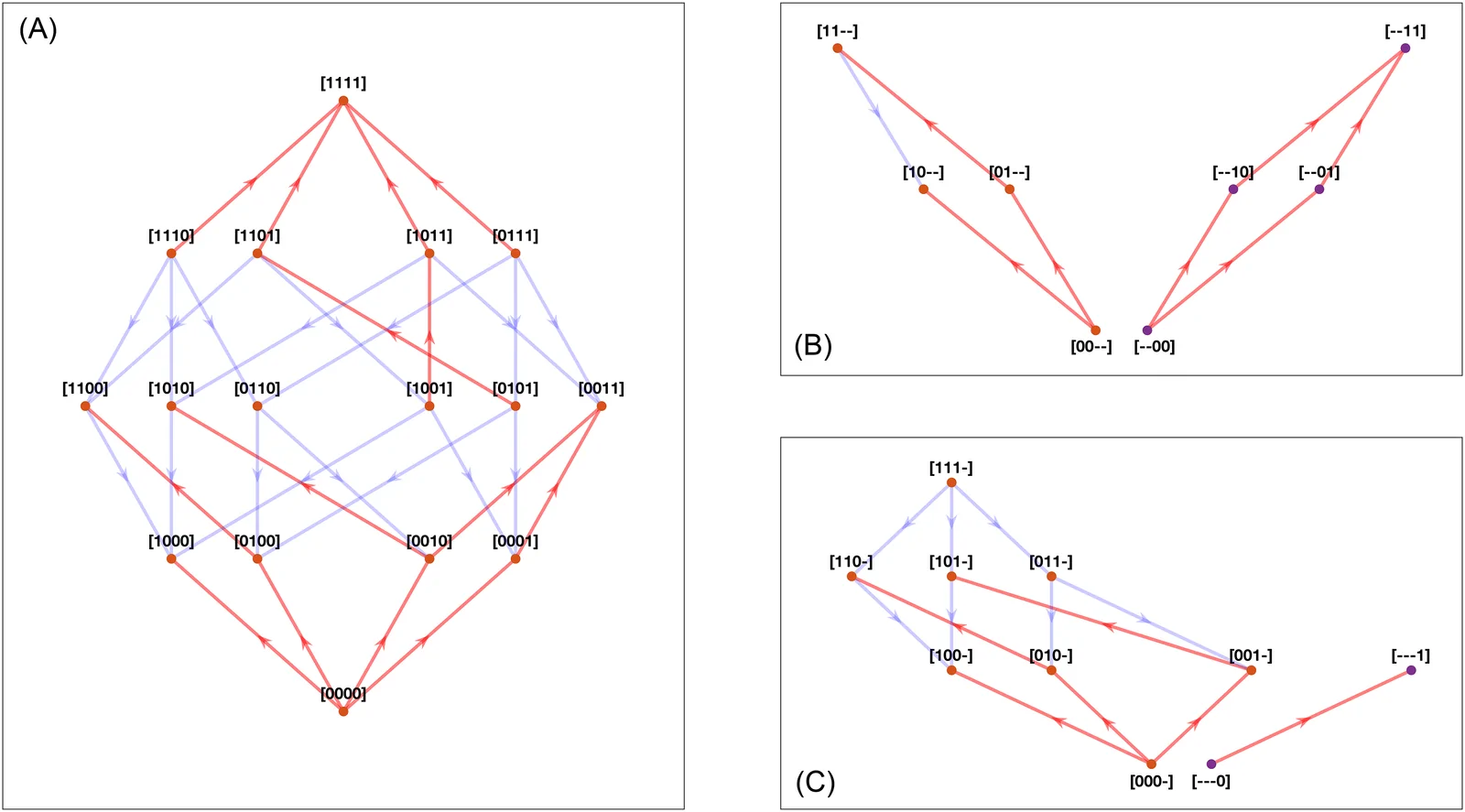
**(A)** An instance of a House of Cards fitness landscape with fitness values assigned by a pseudorandom number generator. Red arrows indicate that the move between the origin node and the single mutant neighbor is possible since the neighbor has higher fitness, while blue arrows indicate that the neighbor has lower fitness and the path is inaccessible. The full landscape is inaccessible. (B) and (C) show two decompositions selected from a total of 15 possible decompositions where the decomposition shown in (B) allows the population to reach the global optimum through combination of the module endpoints. The decomposition shown in (C) does not.

Each step on the modular landscapes is equally likely to be a local optimum in the HoC model, and so we can calculate both the number of possible modular decompositions and the probability that these will produce landscapes that allow for global optimization (e.g., Fig 2B) or curtail adaptation (e.g., Fig 2C).

### Simulations

#### Finding paths through subgraph decompositions.

To find accessible paths through the cultural fitness landscapes, we wrote code in Matlab 2020a (available at https://github.com/LaurelFogarty/CulturalLandscapes) that conducts a depth-first search of the landscapes and their decompositions. First, the code generates a fitness landscape as a nested list of fitness values drawn from a continuous uniform random distribution on (0,1), ensuring that the final type is a global maximum, to provide a House of Cards landscape. By default the landscape is unconstrained, but we can also enforce that the initial type is a global minimum in the constrained HoC case. Using the function ‘oneperm’ [48] the code searches all possible paths through each landscape, one at a time, avoiding the computational expense that would be involved in generating all possible paths simultaneously. The paths are represented as a sequence of numbers which determine the order in which mutations (or innovations) occur. For example in a three component landscape one pathway might be given by the vector {1,2,3}. This indicates a path across the landscape that looks like the following: starting from [0], the first mutational change occurs at position 1 leading to the type **[100]**, the second occurs at position 2 leading to **[110]**, and the final change leads to **[111]**. From here, the code can generate a list of fitness values that occur along the pathway, and check if these values are monotonically increasing.

For each landscape, using similar methods the code can search all possible paths through its decompositions to check whether the endpoints of all the module landscapes in the decomposition are accessible. Thus the code can identify whether the global optimum is accessible in the full landscape or via decomposition and combination.

By ‘combination’ here we mean the following: we take, for example, the landscape shown in Fig 2A and decompose it as shown in Fig 2B. Now, part of the population can search the left-hand decomposition, following beneficial single changes in the components to attempt to reach the endpoint of their module, here**[11- -]**. Similarly, another part of the population can search the right-hand module and attempt to reach their endpoint **[- -11]**. (Alternatively the whole population could search the two modules sequentially.) If the endpoints can be reached they can be ‘recombined’ or communicated to form the global optimum **[1111]** of the full landscape.

However, the search on a module landscape may not reach this endpoint, instead becoming stuck at a local module optimum. In this case recombination may not yield the global optimum on the full landscape as can be seen by considering a search of the decomposition shown in Fig 2C). Conversely, the module endpoint can be reachable without being the optimum in the module landscape, as can be seen in the left-hand module of Fig 2B.

## Results

### Decomposition as a path-finding strategy

Decomposition is advantageous for all sizes of landscape investigated, in that the probability of finding a path to the global optimum through decomposition and combination is higher for all values of *N* that we were able to test (compare black and red solid thick lines in Fig 3). However, this is useful only if it is feasible to search all possible modules. As the number of components increases, the number of possible decompositions increases extremely rapidly and searching all decompositions may become infeasible for even moderately complex technologies. Searching a fixed, smaller number of decompositions may be more manageable when time, energy, or costs are constraints. We calculated the probability of reaching a global optimum when only a small fraction of possible decompositions, chosen at random, can be searched. Here, the search on a modular landscape can be less successful and, especially for larger landscapes and smaller numbers of decompositions searched, it may be less successful than searching the full landscape. A threshold number of searches exists above which decomposition is beneficial. Notably, this number is relatively low. For a 7-component landscape searching 2,4, or 6 decompositions is less successful than searching the full landscape. However, searching 10 decompositions out of a possible 877 (Fig 3 purple line) is sufficient to make decomposition a more successful strategy. Successful decompositions are characterised in Fig 4 in terms of the number of modules and size of the largest module in the decomposition.

**Fig 3:**
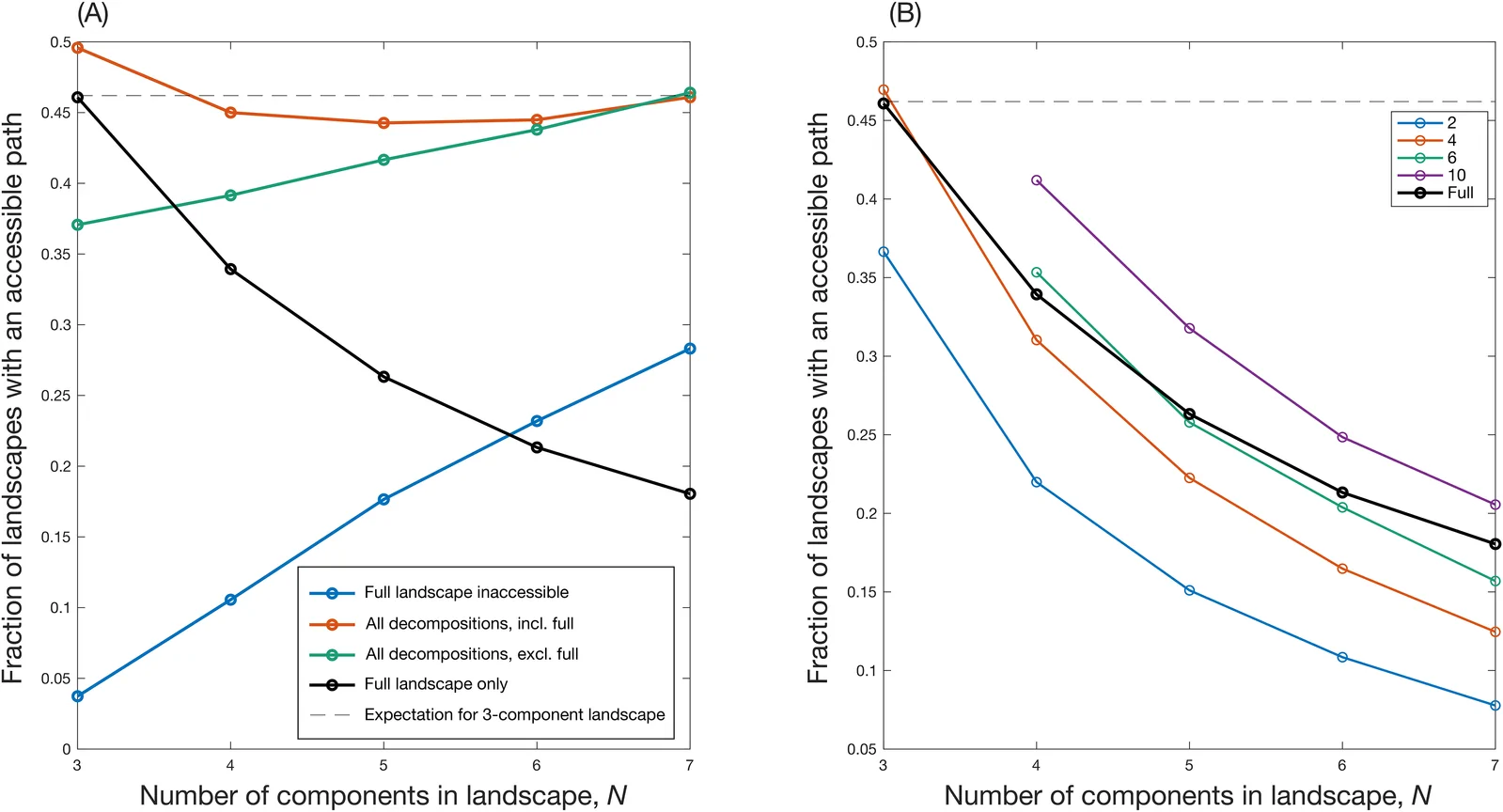
**(A)** Fraction of unconstrained House-of-Cards landscapes where an accessible path to the global maximum was found without decomposition (thick black line) and searching all possible decompositions (thick red line), searching all decompositions excluding the full landscape (green line), and decomposing only landscapes where there is no accessible path through the full landscape (blue line). These are calculated by empirical search of a random ensemble of 5000 landscapes. This is shown for 3,4,5,6 and 7-component landscapes. **(B)** Thin colored lines show the fraction of landscapes for which an accessible path was found when searching just 2, 4, 6, or 10 of the possible decompositions chosen at random.

**Fig 4:**
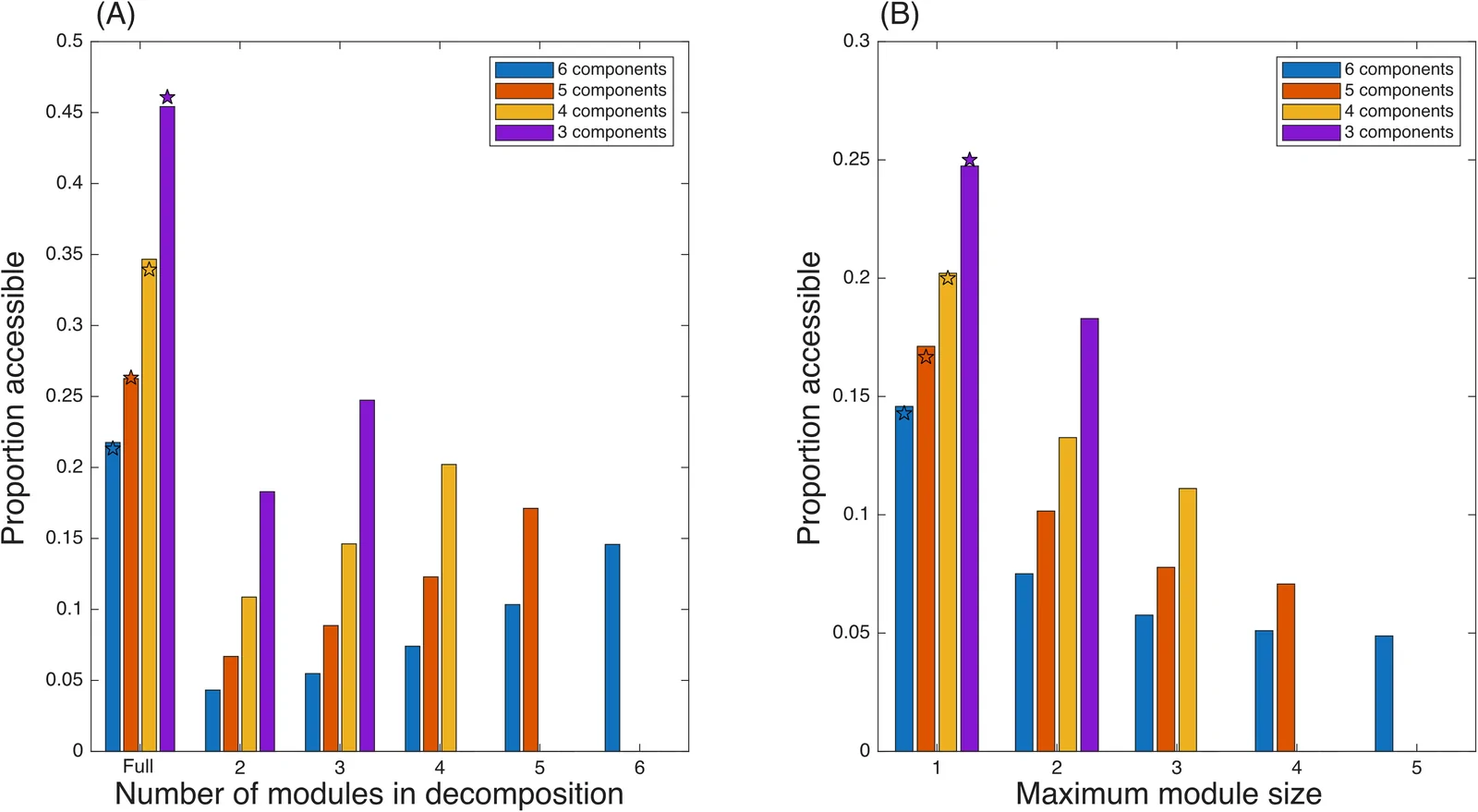
The fraction of unconstrained House-of-Cards landscape decompositions that provide an accessible path to the optimum, separated by (A) the number of modules in the decomposition and (B) the largest module size. In other words, for panel (A) the bar at 2 shows the fraction of 2-module decompositions that provide a path to the optimum for 3 (purple), 4 (yellow), 5 (red) and 6 (blue) component landscapes. In panel (B) the bars at 2 show the fraction of decompositions where the largest module is of size 2. In panel **(B)** It is possible to calculate the values exactly for the case where the largest module has size 1 this is 1/(*n* + 1). These values are marked with stars for comparison to the relevant bar. The bars labeled ‘Full’ in panel (A) are comparable to the black line from Fig 3.

It is also possible to investigate whether landscape decomposition provides an advantage in finding complete paths if only a few possible *paths* in each landscape are searched. Here we consider the case where searchers are willing to search a fixed number, $N_{p}$, of paths through each landscape. The maximum effort to search a path occurs when the whole path must be searched before finding it accessible or inaccessible and so it is reasonable to assume it is proportional to the path length. Thus the maximum effort of searching $N_{p}$ paths of the full landscape is similar to the maximum effort of searching $N_{p}$ paths of each of the module landscapes of a single decomposition. However, searching all the possible decompositions will take proportionally more effort. Fig 5A shows the fraction of successful searches of full and decomposed landscapes in this case for $N_{p}=3$. Searching the decomposed landscapes results in a much better chance of finding a path to the global maximum. However, the total number of searches of the decomposed landscapes will be much greater as the size of the full landscape increases, because the number of possible decompositions is greater. If instead we choose to search only one randomly selected decomposition then we see that there is still an advantage of decomposition for large landscapes, but it is much less dramatic (Fig 5B).

**Fig 5:**
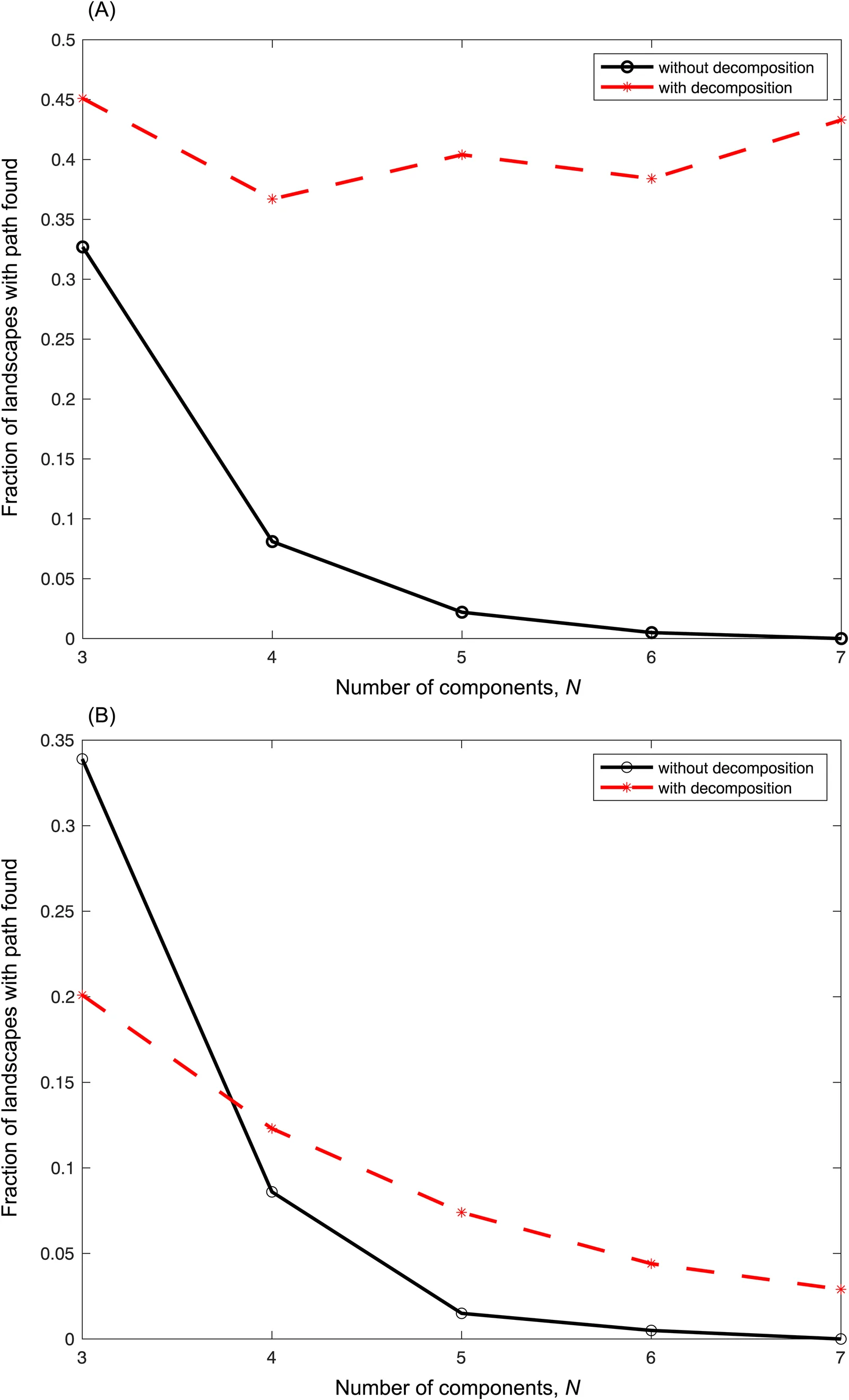
Fraction of unconstrained House-of-Cards landscapes where a path to the global maximum was found successfully by (A) searching 3 paths through each landscape without decomposition (black line) and by searching 3 paths through the full landscape and all its possible decompositions (red dashed line), (B) searching 3 paths through each landscape without decomposition (black line) and by searching 3 paths through each module of a single randomly selected decomposition (red dashed line). These are calculated by empirical search of a random ensemble of 1000 landscapes, shown for 3,4,5,6 and 7-component landscapes.

### The time and communication cost of decomposition

In cultural contexts, the time taken to cross cultural fitness landscapes can be critically important. Turning again to the example of cassava detoxification, in times of famine the health costs of eating a small amount of cassava that has not been fully detoxified might be heavily outweighed by the costs of not eating at all. In this case, calculations of the ‘fitness’ of certain solutions can shift; the best solution is now the fastest. Similarly, in the context of technological development, communication between team members working on different aspects of a problem can create a heavy time burden that can derail collaborative projects entirely [49].

From Fig 3 it is clear that decomposing landscapes and searching those decompositions increases the probability that a landscape is accessible. However, to begin to understand how the time taken to search the landscapes in their decomposed forms might change with the number of decompositions, we calculate the computational search time in a number of cases. For an ensemble of 1000 landscapes, we calculate the time taken to search the full landscape until an accessible path is found or all paths have been searched, and the same for the decomposed landscape. We benchmark just the function that checks accessibility and see that it is more time-consuming to decompose the landscape and search the decompositions (excluding the full landscape) than it is to search the full landscape (Fig 6), although this may change with the number of parallel searches.

**Fig 6:**
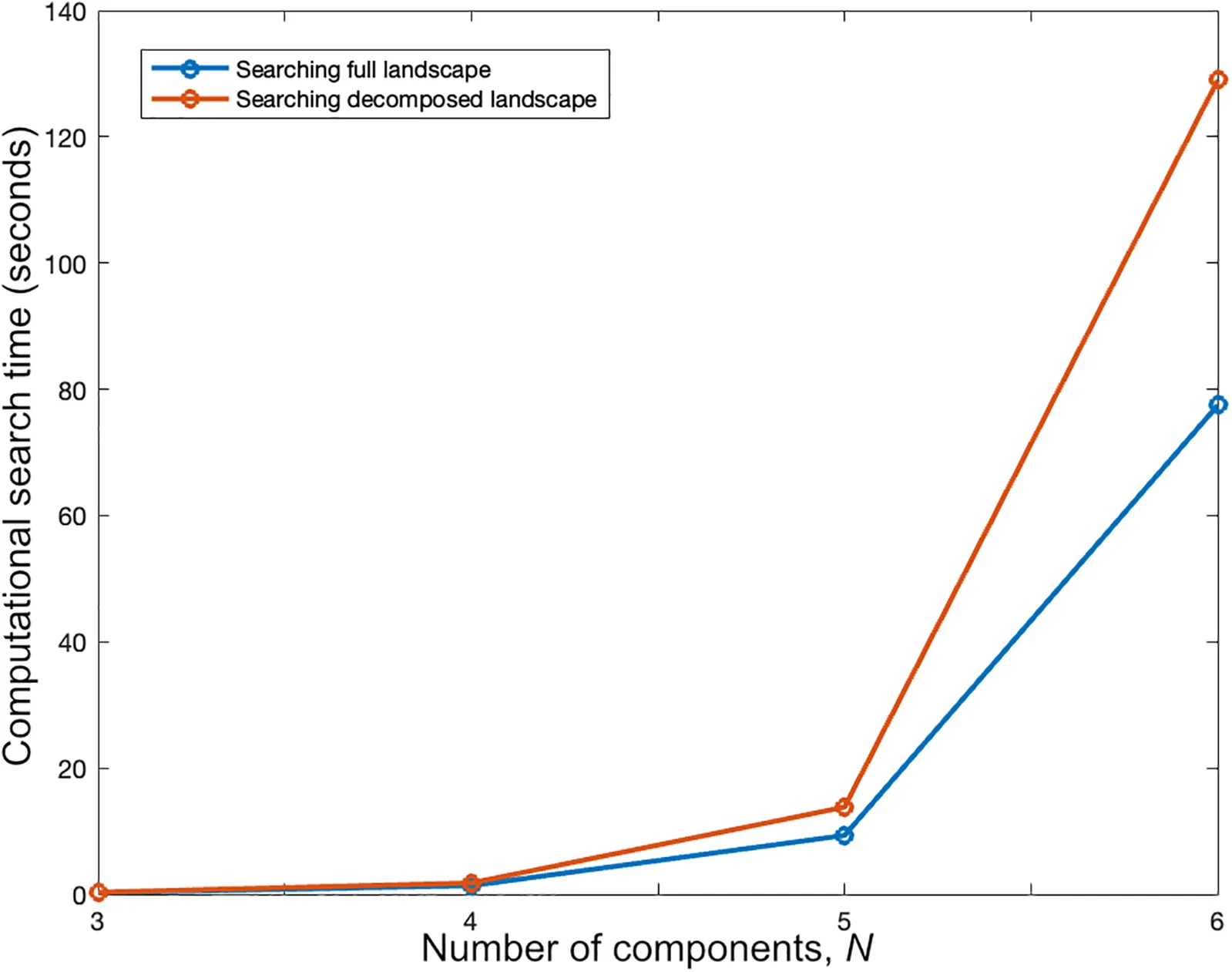
Computational time taken to search 1000 full landscapes or 1000 decomposed landscapes until an accessible path or decomposition is found or the landscape is found to be inaccessible.

This exercise gives us some indication of time costs, but does not account for communication costs. A rough re-interpretation of what is known as ‘Brook’s law’ [49], might allow us to make an estimate of this cost. Brooks suggested his ‘law’ originally to address the organization of personnel on software projects. He suggested that “Adding manpower to a late software project makes it later.” This was considered to be a direct result of training and communication costs. The communication costs between *m* individuals were formulated roughly as m(m−1)/2 where *m* is the number of individuals communicating pairwise. In our context, if one individual or group searches one decomposition and then communicates the results, such a formula would suggest that communications costs can increase very rapidly - the costs of communicating the search results for 10 decompositions would be 45 times that of communicating the results from 2.

Thus we expect the costs of searching landscape decompositions and recombining results to trade off against the greater likelihood of a successful search. Whether decomposition and recombination is the more successful strategy depends on how great the costs are relative to the accessibility advantage, which as the cassava example illustrates, will be strongly context-dependent.

## Discussion

We aimed to construct a series of models of cultural fitness landscapes in which the unique dynamics of cultural and technological evolution could be better understood. In particular, we focused on modularization, collaboration and social learning, and efficiency trade-offs - three critical components of technological evolution across human cultural history [17,18,50].

We showed that modularization in combination with collaborative communication of search results was always more successful (but sometimes more costly) than searching a full landscape for all the ‘numbers of components’ (values of *N*) in the landscapes that we tested. However, this may not be generally true. In the genetic case [13], showed that for most landscape types, populations traversing landscapes with a very small or a very large number of components were typically more likely to reach the global optimum than those traversing intermediate sized landscapes. This may mean that for very large technological landscapes (or, equivalently, very complex technologies) the relationship between the costs and benefits of modularization may be altered. The number of possible decompositions in such cases makes analysis of large landscapes computationally challenging, but for real populations, completely infeasible. For example, for the HoC model a 20 component landscape has 5.1724e + 13 possible decompositions. As a demonstration of this point, it is also possible to calculate the computational time taken to search such landscapes (see Fig 6). In our cassava example (Fig 1) examining or trying this number of combinations would be impossible within a reasonable number of years or even generations, not to mention a dangerous undertaking.

Indeed, in many of the circumstances under which technology evolves, a full search of many thousands of possible decompositions may not be feasible and time, financial, energetic, and environmental constraints are likely to be strong [50]. Therefore, it is important to ask – what is the the probability of successfully finding a global optimum when only a small number of decompositions can be examined? Our analysis suggests that there is a critical value for the number of searchable decompositions below which the global optimum is more likely to be reached by searching the full landscape. The value of this threshold depends on the complexity of the technology (in other words, on the number of components in the technological landscape, *N*).

Another similar constraint may exist on the number of paths, rather than decompositions, that can be investigated. If the search time is greater for decomposed landscapes, as it is if a fixed number of paths is searched and the expected search time of each path is a constant, then, intuitively, there is no advantage in decomposing a landscape and the probability of successful searches using such a limited effort strategy is low (Fig 5B).

It is possible to consider, too, the unique timescale on which technology or other cultural traits can evolve. Searches of modules, in other words efforts to improve particular parts of a technology, might happen at quite different times in human history. Such findings might be recorded, and then communicated and incorporated into existing technology at times that could be separated on a scale of years, decades or centuries. Many compelling examples of this type of time separation exist in the history of human culture including the rediscovery of Gregor Mendel’s work on inheritance over thirty years after its publication, which laid the foundations of modern evolutionary theory. This possibility might considerably alter the types of constraints that searches on cultural landscapes might be subject to, for example by allowing searches to take place when costs allow.

In the search for optimal technological designs, these models suggest that the trade-offs between decomposition, costs of searching, and costs of communication must be carefully considered and that the relative benefits and costs of these are often unintuitive. We have not discussed, however, the structure of the landscapes themselves. We presented results for House of Cards landscapes, which, while mathematically expedient have been shown to be less relevant in genetically evolving systems than other landscape structures [13]. Considerable further work focused on establishing the exact structure of cultural fitness landscapes and on understanding the ways in which these are, or could be, decomposed and searched most efficiently remains to be done.

## Supporting information

S1 File Pseudocode. This file contains pseudocode describing the algorithms that produced the results described in the paper.
(PDF)
