# Supplementary material for: The evolutionary accessibility of cultural fitness landscapes
Source: PLoS Comput Biol. 2026 Oct 22;22(10):e1014830. doi: 10.1371/journal.pcbi.1014830 (PMC13722292; doi:10.1371/journal.pcbi.1014830)
Supplement: Supplementary file 1 [file pcbi.1014830.s001.pdf]

Supplementary information for: The evolutionary accessibility of cultural fitness landscapes

Laurel Fogarty[1,*], Rebecca B. Hoyle[2],

1 Department of Human Behavior, Ecology, and Culture, Max Planck Institute for Evolutionary Anthropology, 04103 Leipzig, Germany
2 School of Mathematical Sciences, University of Southampton, Southampton, UK

* laurel_fogarty@eva.mpg.de

S1: Pseudocode

Algorithm 1 Estimating Accessibility of Fitness Landscapes and Their Decompositions

```
Require: Number of replicates R = 5000; component counts C = [3, 4, 5, 6, 7]
Require: Constraint flag constrained ∈ {0, 1}
Ensure: Probabilities probNoPath[g], probNoDecompPath[g]
for each number i of genes n in C do
    Initialise complete_path[1..R] ← 0
    Initialise decomp_complete_path[1..R] ← 0
    Generate genotype set for n components
    Enumerate all decompositions of the n-component landscape
    for each replicate r = 1 to R do
        Assign random fitness values to all genotypes
        Ensure all-1s genotype is the global maximum
        if constrained = 1 then
            Ensure all-0s genotype is the global minimum
        end if
        for each decomposition D of the landscape do
            complete_through_decomp ← true
            for each sub-landscape L in D do
                Extract components and fitness values for L
                if FindPath(L) = false then
                    complete_through_decomp ← false
                    break
                end if
            end for
            if complete_through_decomp then
                decomp_complete_path[r] ← 1
            end if
        end for
    end for
    probNoPath[i] ← 1 − mean(complete_path)
    probNoDecompPath[i] ← 1 − mean(decomp_complete_path)
end for
Save probNoPath and probNoDecompPath
```

Algorithm 2 FindPath: Determine whether a complete adaptive path exists through a fitness landscape

Require: Number of components n, fitness values *fitness* for each genotype
Ensure: Boolean variable *pathcomplete* indicating if any monotonic path exists

```
pathcomplete ← 0
for p = 1 to factorial(n) do                    ▷ Iterate over all possible mutation orders
    paths ← oneperm(n, p)
    Initialise fitness_value[1] ← fitness(1)
    for q = 1 to n do
        C ← nchoosek(1:n, q)
        genotypes ← binary matrix with ones at positions in C
        path ← zero vector of length n
        path[paths(1:q)] ← 1
        fitnessIndex ← find row(path) in (genotypes)
        fitness_value[q + 1] ← fitness{q + 1}[fitnessIndex(1)]
    end for
    DIFF ← calculate differences between adjacent elements(fitness_value)
    Remove zero entries from DIFF
    if sum(|DIFF| = DIFF) = n then
        pathcomplete ← 1
        break
    end if
end for
return pathcomplete
```
